# 1-(2-Bromo­acet­yl)-3-methyl-2,6-diphenyl­piperidin-4-one

**DOI:** 10.1107/S160053681001901X

**Published:** 2010-05-29

**Authors:** G. Aridoss, S. Sundaramoorthy, D. Velmurugan, K. S. Park, Y. T. Jeong

**Affiliations:** a Department of Image Science and Engineering, Pukyong National University, Busan 608-739, Republic of Korea; b Centre of Advanced Study in Crystallography and Biophysics, University of Madras, Guindy Campus, Chennai 600 025, India

## Abstract

In the title compound, C_20_H_20_BrNO_2_, the piperidone ring adopts a boat conformation. The phenyl rings are oriented at dihedral angles of 97.8 (2) and 96.0 (1)° with respect to the best plane through the piperidine ring. The dihedral angle between the two phenyl rings is 49.7 (1)°. In the crystal, bifurcated C—H⋯O hydrogen bonds form a *R*_2_^1^(7) ring motif, linking the mol­ecules into centrosymmetric dimers.

## Related literature

For the biological activity of functionalized piperidines, see: Richardo *et al.* (1979 ▶); Schneider (1996 ▶); Mukhtar & Wright (2005 ▶); Aridoss *et al.* (2007 ▶); Winkler & Holan (1989 ▶). For related structures see: Aridoss *et al.* (2009*a* ▶,*b* ▶). For ring conformational analysis, see: Cremer & Pople (1975 ▶); Nardelli (1983 ▶).
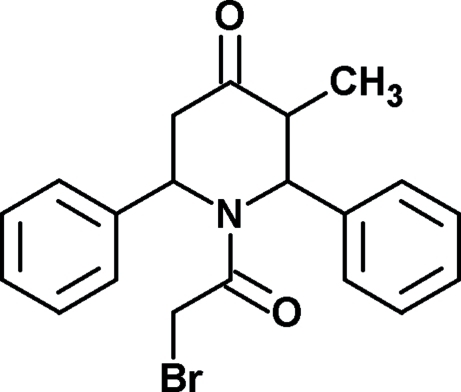


## Experimental

### 

#### Crystal data

C_20_H_20_BrNO_2_
*M* *_r_* = 386.28
Monoclinic, 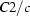

*a* = 21.4006 (8) Å
*b* = 14.5873 (6) Å
*c* = 13.8107 (5) Å
β = 125.368 (2)°
*V* = 3515.7 (2) Å^3^
*Z* = 8
Mo *K*α radiation
μ = 2.35 mm^−1^
*T* = 292 K
0.3 × 0.26 × 0.22 mm

#### Data collection

Bruker SMART APEXII area-detector diffractometer
Absorption correction: multi-scan (*SADABS*; Bruker, 2008 ▶) *T*_min_ = 0.499, *T*_max_ = 0.596
17094 measured reflections
4398 independent reflections
2725 reflections with *I* > 2σ(*I*)
*R*_int_ = 0.035

#### Refinement

*R*[*F* ^2^ > 2σ(*F* ^2^)] = 0.059
*wR*(*F* ^2^) = 0.213
*S* = 1.02
4398 reflections
218 parameters
H-atom parameters constrained
Δρ_max_ = 0.53 e Å^−3^
Δρ_min_ = −0.77 e Å^−3^

### 

Data collection: *APEX2* (Bruker, 2008 ▶); cell refinement: *SAINT* (Bruker, 2008 ▶); data reduction: *SAINT*; program(s) used to solve structure: *SHELXS97* (Sheldrick, 2008 ▶); program(s) used to refine structure: *SHELXL97* (Sheldrick, 2008 ▶); molecular graphics: *ORTEP-3* (Farrugia, 1997 ▶); software used to prepare material for publication: *SHELXL97* and *PLATON* (Spek, 2009 ▶).

## Supplementary Material

Crystal structure: contains datablocks global, I. DOI: 10.1107/S160053681001901X/bt5275sup1.cif

Structure factors: contains datablocks I. DOI: 10.1107/S160053681001901X/bt5275Isup2.hkl

Additional supplementary materials:  crystallographic information; 3D view; checkCIF report

## Figures and Tables

**Table 1:** Hydrogen-bond geometry (Å, °)

*D*—H⋯*A*	*D*—H	H⋯*A*	*D*⋯*A*	*D*—H⋯*A*
C2—H2*A*⋯O1^i^	0.97	2.56	3.458 (6)	154
C13—H13⋯O1^i^	0.93	2.51	3.404 (5)	161

Symmetry code: (i) 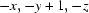.

**Fig. 1.**
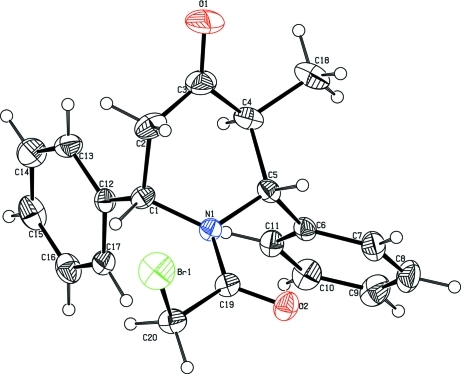
Perspective view of the molecule showing the thermal ellipsoids are drawn at 30% probability level.

**Fig. 2.**
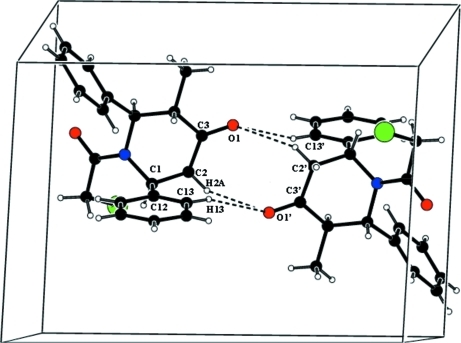
Crystal packing showing the formation of ring R21(7) Bifurcated and the centrosymmetric R22(8) dimer. For the clarity, H atoms are deleted which are not involved in the bond formation.

## supplementary crystallographic information

### Comment

Amides are prominent functional groups in chemistry due to their integral part in biologically important polymers such as peptides and proteins. Functionalized piperidines are among the most common building blocks in natural products and more interestingly, in many biologically active compounds such as anopterine, pergoline, scopolamine and morphine (Richardo *et al.*, 1979, Schneider, 1996, Mukhtar & Wright, 2005). Piperidones also have high impact in medicinal field owing to their role as key chiral intermediates for the preparation of a variety of natural, synthetic and semi-synthetic pharmacophores with marked anticancer  and anti-HIV activities (Winkler & Holan, 1989). It has been established by our earlier studies (Aridoss *et al.* 2009a, Aridoss *et al.* 2009b) that unlike the substitution of either alky or aryl system in the carbon skeleton of piperidone, incorporation of either chloroacetyl or bromoacetyl functionality at the nitrogen of piperidone remarkably changes the rigid chair confirmation of heterocyclic ring into non-chair conformation of its preference. Thus to find out the change in conformation of 2,6-diphenyl-3-methylpiperidin-4-one upon bromoacetylation, the title compound was synthesized and discussed here with its X-ray crystallographic data.

In the present structure, the piperidone ring adopts a boat conformation with atoms C1 and C4 deviating by 0.395 (1) and 0.334 (1) Å, respectively, from the least-sqaures plane defined by the remaining atoms (N1/C2/C3/C5) in the ring. When compared with the reported structures of piperidone derivatives (Aridoss *et al.*, 2009b), it is clear that the conformation of the piperidone ring is highly influenced by the substitutions at various positions.The sum of the bond angles around the atom N1(357.6 (6)°) of the piperidone ring in the molecule is in accordance with sp^2^ hybridization.

The puckering parameters (Cremer & Pople,1975) and the smallest displacement asymmetry parameters (Nardelli, 1983) for piperidone ring are q_2_ = 0.639 (4) Å, q_3_ = 0.062 (1) Å; Q_T_ = 0.642 (4) Å and φ_2_ = 84.4 (1) °, respectively. Atoms C2 and C13 act as donors to form bifurcated hydrogen bonds with atom O1' as an aceptor. In the crystal structure, the molecules at (x,y,z) and (-x,-y-1, -z ) are linked by C2—H2A···O1' hydrogen bonds into cyclic centrosymmetric R_2_^2^(8) dimer.

### Experimental

The title compound was obtained by adopting our earlier method (Aridoss *et al.* 2007). To a solution of 2,6-diphenyl-3-methylpiperidin -4-one (1 equiv.) and NEt3 (1.5 equiv.) in freshly distilled benzene, bromoacetyl chloride (1 equiv.) in benzene was added in drop wise. After the completion of reaction, the crude compound was obtained by evaporation of its ethyl acetate extract. This upon recrystallization in distilled ethanol afforded fine white crystals suitable for X-ray diffraction study.

### Refinement

H atoms were positioned geometrically (C—H=0.93-0.98Å) and allowed to ride on their parent atoms, with 1.5U_eq_(C) for methyl H and 1.2 U_eq_(C) for other H atoms.

### Crystal data

**Table:**

C_20_H_20_BrNO_2_	*F*(000) = 1584
*M**_r_* = 386.28	*D*_x_ = 1.460 Mg m^−^^3^
Monoclinic, *C*2/*c*	Mo *K*α radiation, λ = 0.71073 Å
Hall symbol: -C 2yc	Cell parameters from 2025 reflections
*a* = 21.4006 (8) Å	θ = 0.5–0.6°
*b* = 14.5873 (6) Å	µ = 2.35 mm^−^^1^
*c* = 13.8107 (5) Å	*T* = 292 K
β = 125.368 (2)°	Block, colorless
*V* = 3515.7 (2) Å^3^	0.3 × 0.26 × 0.22 mm
*Z* = 8	

### Data collection

**Table:**

Bruker SMART APEXII area-detector diffractometer	4398 independent reflections
Radiation source: fine-focus sealed tube	2725 reflections with *I* > 2σ(*I*)
graphite	*R*_int_ = 0.035
ω and φ scans	θ_max_ = 28.5°, θ_min_ = 1.8°
Absorption correction: multi-scan (*SADABS*; Bruker, 2008)	*h* = −27→28
*T*_min_ = 0.499, *T*_max_ = 0.596	*k* = −19→19
17094 measured reflections	*l* = −18→18

### Refinement

**Table:**

Refinement on *F*^2^	Primary atom site location: structure-invariant direct methods
Least-squares matrix: full	Secondary atom site location: difference Fourier map
*R*[*F*^2^ > 2σ(*F*^2^)] = 0.059	Hydrogen site location: inferred from neighbouring sites
*wR*(*F*^2^) = 0.213	H-atom parameters constrained
*S* = 1.02	*w* = 1/[σ^2^(*F*_o_^2^) + (0.1259*P*)^2^ + 4.1414*P*] where *P* = (*F*_o_^2^ + 2*F*_c_^2^)/3
4398 reflections	(Δ/σ)_max_ < 0.001
218 parameters	Δρ_max_ = 0.53 e Å^−^^3^
0 restraints	Δρ_min_ = −0.77 e Å^−^^3^

### Special details

**Table:**

Geometry. All esds (except the esd in the dihedral angle between two l.s. planes) are estimated using the full covariance matrix. The cell esds are taken into account individually in the estimation of esds in distances, angles and torsion angles; correlations between esds in cell parameters are only used when they are defined by crystal symmetry. An approximate (isotropic) treatment of cell esds is used for estimating esds involving l.s. planes.
Refinement. Refinement of *F*^2^ against ALL reflections. The weighted *R*-factor wR and goodness of fit *S* are based on *F*^2^, conventional *R*-factors *R* are based on *F*, with *F* set to zero for negative *F*^2^. The threshold expression of *F*^2^ > σ(*F*^2^) is used only for calculating *R*-factors(gt) etc. and is not relevant to the choice of reflections for refinement. *R*-factors based on *F*^2^ are statistically about twice as large as those based on *F*, and *R*- factors based on ALL data will be even larger.

### Fractional atomic coordinates and isotropic or equivalent isotropic displacement parameters (Å^2^)

**Table:**

	*x*	*y*	*z*	*U*_iso_*/*U*_eq_	
C1	0.00908 (18)	0.2545 (2)	0.0494 (3)	0.0355 (7)	
H1	0.0555	0.2317	0.1229	0.043*	
C2	0.0317 (2)	0.3418 (3)	0.0168 (4)	0.0517 (10)	
H2A	0.0432	0.3889	0.0745	0.062*	
H2B	0.0780	0.3303	0.0214	0.062*	
C3	−0.0287 (3)	0.3764 (3)	−0.1039 (4)	0.0581 (11)	
C4	−0.0933 (2)	0.3111 (3)	−0.1867 (3)	0.0463 (8)	
H4	−0.1307	0.3148	−0.1674	0.056*	
C5	−0.06703 (18)	0.2104 (2)	−0.1692 (3)	0.0359 (7)	
H5	−0.0393	0.2039	−0.2056	0.043*	
C6	−0.13640 (19)	0.1469 (2)	−0.2373 (3)	0.0355 (7)	
C7	−0.1502 (2)	0.0969 (3)	−0.3328 (3)	0.0502 (9)	
H7	−0.1150	0.0987	−0.3518	0.060*	
C8	−0.2159 (3)	0.0440 (3)	−0.4008 (3)	0.0605 (11)	
H8	−0.2249	0.0115	−0.4657	0.073*	
C9	−0.2672 (2)	0.0396 (3)	−0.3726 (4)	0.0622 (12)	
H9	−0.3110	0.0038	−0.4179	0.075*	
C10	−0.2541 (2)	0.0881 (3)	−0.2772 (4)	0.0526 (10)	
H10	−0.2891	0.0848	−0.2577	0.063*	
C11	−0.1889 (2)	0.1421 (2)	−0.2096 (3)	0.0408 (8)	
H11	−0.1805	0.1752	−0.1455	0.049*	
C12	−0.05143 (17)	0.2656 (2)	0.0741 (3)	0.0334 (7)	
C13	−0.0720 (2)	0.3505 (2)	0.0908 (4)	0.0470 (9)	
H13	−0.0510	0.4034	0.0831	0.056*	
C14	−0.1253 (3)	0.3567 (3)	0.1196 (4)	0.0584 (11)	
H14	−0.1388	0.4140	0.1315	0.070*	
C15	−0.1573 (2)	0.2800 (3)	0.1301 (4)	0.0566 (11)	
H15	−0.1934	0.2850	0.1469	0.068*	
C16	−0.1354 (2)	0.1944 (3)	0.1156 (3)	0.0508 (9)	
H16	−0.1566	0.1417	0.1233	0.061*	
C17	−0.0820 (2)	0.1873 (3)	0.0896 (3)	0.0408 (8)	
H17	−0.0665	0.1297	0.0825	0.049*	
C18	−0.1349 (3)	0.3390 (3)	−0.3166 (5)	0.0706 (14)	
H18A	−0.1495	0.4023	−0.3255	0.106*	
H18B	−0.1800	0.3019	−0.3648	0.106*	
H18C	−0.1016	0.3302	−0.3412	0.106*	
C19	0.02886 (17)	0.1079 (2)	−0.0205 (3)	0.0353 (7)	
C20	0.09463 (19)	0.0844 (3)	0.1063 (3)	0.0432 (8)	
H20A	0.1063	0.0195	0.1125	0.052*	
H20B	0.0803	0.0982	0.1598	0.052*	
N1	−0.01254 (14)	0.18526 (19)	−0.0422 (2)	0.0328 (6)	
O1	−0.0265 (3)	0.4523 (2)	−0.1361 (4)	0.1037 (15)	
O2	0.01584 (14)	0.05669 (18)	−0.1000 (2)	0.0472 (6)	
Br1	0.18249 (2)	0.15486 (4)	0.14906 (5)	0.0736 (3)	

### Atomic displacement parameters (Å^2^)

**Table:**

	*U*^11^	*U*^22^	*U*^33^	*U*^12^	*U*^13^	*U*^23^
C1	0.0316 (14)	0.0321 (16)	0.0404 (17)	−0.0039 (12)	0.0195 (13)	−0.0042 (13)
C2	0.048 (2)	0.040 (2)	0.076 (3)	−0.0124 (15)	0.041 (2)	−0.0081 (18)
C3	0.080 (3)	0.041 (2)	0.074 (3)	−0.008 (2)	0.056 (3)	0.003 (2)
C4	0.050 (2)	0.0399 (19)	0.055 (2)	0.0067 (16)	0.0333 (18)	0.0140 (17)
C5	0.0375 (16)	0.0403 (18)	0.0344 (16)	0.0051 (13)	0.0234 (14)	0.0063 (14)
C6	0.0326 (15)	0.0386 (17)	0.0287 (14)	0.0096 (12)	0.0140 (13)	0.0056 (13)
C7	0.051 (2)	0.059 (2)	0.0373 (18)	0.0107 (18)	0.0239 (17)	0.0013 (17)
C8	0.059 (2)	0.061 (3)	0.0353 (18)	0.009 (2)	0.0120 (17)	−0.0110 (18)
C9	0.042 (2)	0.058 (3)	0.050 (2)	−0.0027 (18)	0.0060 (17)	−0.011 (2)
C10	0.0347 (18)	0.061 (3)	0.054 (2)	0.0003 (16)	0.0205 (16)	−0.0042 (19)
C11	0.0352 (16)	0.046 (2)	0.0372 (17)	0.0049 (14)	0.0187 (14)	−0.0013 (15)
C12	0.0284 (13)	0.0383 (17)	0.0270 (14)	−0.0006 (12)	0.0124 (12)	−0.0013 (13)
C13	0.053 (2)	0.0346 (18)	0.054 (2)	0.0059 (15)	0.0313 (19)	0.0036 (16)
C14	0.066 (3)	0.057 (3)	0.064 (3)	0.021 (2)	0.044 (2)	0.007 (2)
C15	0.043 (2)	0.086 (3)	0.046 (2)	0.013 (2)	0.0288 (17)	0.009 (2)
C16	0.047 (2)	0.064 (2)	0.0411 (19)	−0.0131 (19)	0.0259 (17)	−0.0015 (18)
C17	0.0459 (19)	0.0381 (18)	0.0377 (17)	−0.0052 (15)	0.0237 (15)	−0.0045 (15)
C18	0.077 (3)	0.066 (3)	0.063 (3)	0.011 (2)	0.037 (3)	0.030 (2)
C19	0.0279 (14)	0.0363 (17)	0.0400 (17)	0.0017 (12)	0.0187 (13)	0.0006 (14)
C20	0.0322 (15)	0.0412 (19)	0.0468 (19)	0.0022 (13)	0.0175 (14)	0.0030 (15)
N1	0.0313 (13)	0.0331 (13)	0.0329 (13)	0.0013 (10)	0.0179 (11)	−0.0002 (11)
O1	0.161 (4)	0.047 (2)	0.096 (3)	−0.030 (2)	0.071 (3)	0.0106 (18)
O2	0.0424 (13)	0.0498 (15)	0.0456 (14)	0.0084 (11)	0.0233 (11)	−0.0064 (12)
Br1	0.0406 (3)	0.0898 (5)	0.0780 (4)	−0.0045 (2)	0.0272 (3)	−0.0027 (3)

### Geometric parameters (Å, °)

**Table:**

C1—N1	1.468 (4)	C10—C11	1.391 (5)
C1—C2	1.521 (5)	C10—H10	0.9300
C1—C12	1.527 (4)	C11—H11	0.9300
C1—H1	0.9800	C12—C13	1.378 (5)
C2—C3	1.486 (7)	C12—C17	1.394 (5)
C2—H2A	0.9700	C13—C14	1.411 (6)
C2—H2B	0.9700	C13—H13	0.9300
C3—O1	1.204 (5)	C14—C15	1.362 (7)
C3—C4	1.516 (6)	C14—H14	0.9300
C4—C18	1.525 (6)	C15—C16	1.389 (6)
C4—C5	1.541 (5)	C15—H15	0.9300
C4—H4	0.9800	C16—C17	1.384 (5)
C5—N1	1.485 (4)	C16—H16	0.9300
C5—C6	1.527 (5)	C17—H17	0.9300
C5—H5	0.9800	C18—H18A	0.9600
C6—C7	1.382 (5)	C18—H18B	0.9600
C6—C11	1.381 (5)	C18—H18C	0.9600
C7—C8	1.387 (6)	C19—O2	1.219 (4)
C7—H7	0.9300	C19—N1	1.357 (4)
C8—C9	1.362 (7)	C19—C20	1.520 (5)
C8—H8	0.9300	C20—Br1	1.912 (4)
C9—C10	1.375 (6)	C20—H20A	0.9700
C9—H9	0.9300	C20—H20B	0.9700
			
N1—C1—C2	108.5 (3)	C11—C10—H10	119.9
N1—C1—C12	112.4 (2)	C6—C11—C10	120.3 (3)
C2—C1—C12	115.7 (3)	C6—C11—H11	119.8
N1—C1—H1	106.6	C10—C11—H11	119.8
C2—C1—H1	106.6	C13—C12—C17	119.2 (3)
C12—C1—H1	106.6	C13—C12—C1	121.9 (3)
C3—C2—C1	113.4 (3)	C17—C12—C1	118.8 (3)
C3—C2—H2A	108.9	C12—C13—C14	119.5 (4)
C1—C2—H2A	108.9	C12—C13—H13	120.2
C3—C2—H2B	108.9	C14—C13—H13	120.2
C1—C2—H2B	108.9	C15—C14—C13	121.0 (4)
H2A—C2—H2B	107.7	C15—C14—H14	119.5
O1—C3—C2	122.2 (5)	C13—C14—H14	119.5
O1—C3—C4	120.8 (5)	C14—C15—C16	119.4 (4)
C2—C3—C4	117.0 (3)	C14—C15—H15	120.3
C3—C4—C18	112.1 (3)	C16—C15—H15	120.3
C3—C4—C5	112.9 (3)	C17—C16—C15	120.2 (4)
C18—C4—C5	110.5 (3)	C17—C16—H16	119.9
C3—C4—H4	107.0	C15—C16—H16	119.9
C18—C4—H4	107.0	C16—C17—C12	120.6 (4)
C5—C4—H4	107.0	C16—C17—H17	119.7
N1—C5—C6	113.4 (3)	C12—C17—H17	119.7
N1—C5—C4	112.7 (3)	C4—C18—H18A	109.5
C6—C5—C4	110.2 (3)	C4—C18—H18B	109.5
N1—C5—H5	106.7	H18A—C18—H18B	109.5
C6—C5—H5	106.7	C4—C18—H18C	109.5
C4—C5—H5	106.7	H18A—C18—H18C	109.5
C7—C6—C11	118.6 (3)	H18B—C18—H18C	109.5
C7—C6—C5	120.2 (3)	O2—C19—N1	122.1 (3)
C11—C6—C5	121.1 (3)	O2—C19—C20	118.4 (3)
C6—C7—C8	120.8 (4)	N1—C19—C20	119.5 (3)
C6—C7—H7	119.6	C19—C20—Br1	108.9 (2)
C8—C7—H7	119.6	C19—C20—H20A	109.9
C9—C8—C7	120.1 (4)	Br1—C20—H20A	109.9
C9—C8—H8	120.0	C19—C20—H20B	109.9
C7—C8—H8	120.0	Br1—C20—H20B	109.9
C8—C9—C10	120.0 (4)	H20A—C20—H20B	108.3
C8—C9—H9	120.0	C19—N1—C1	122.8 (3)
C10—C9—H9	120.0	C19—N1—C5	115.6 (3)
C9—C10—C11	120.1 (4)	C1—N1—C5	119.2 (3)
C9—C10—H10	119.9		
			
N1—C1—C2—C3	55.3 (4)	C2—C1—C12—C13	−13.3 (5)
C12—C1—C2—C3	−72.0 (4)	N1—C1—C12—C17	46.6 (4)
C1—C2—C3—O1	166.4 (5)	C2—C1—C12—C17	172.0 (3)
C1—C2—C3—C4	−14.4 (5)	C17—C12—C13—C14	−1.9 (5)
O1—C3—C4—C18	19.2 (6)	C1—C12—C13—C14	−176.7 (3)
C2—C3—C4—C18	−159.9 (4)	C12—C13—C14—C15	−0.6 (7)
O1—C3—C4—C5	144.8 (5)	C13—C14—C15—C16	1.8 (7)
C2—C3—C4—C5	−34.4 (5)	C14—C15—C16—C17	−0.5 (6)
C3—C4—C5—N1	41.5 (4)	C15—C16—C17—C12	−2.0 (5)
C18—C4—C5—N1	167.9 (3)	C13—C12—C17—C16	3.2 (5)
C3—C4—C5—C6	169.2 (3)	C1—C12—C17—C16	178.1 (3)
C18—C4—C5—C6	−64.3 (4)	O2—C19—C20—Br1	98.6 (3)
N1—C5—C6—C7	−119.1 (3)	N1—C19—C20—Br1	−80.3 (3)
C4—C5—C6—C7	113.5 (3)	O2—C19—N1—C1	−170.7 (3)
N1—C5—C6—C11	64.7 (4)	C20—C19—N1—C1	8.1 (5)
C4—C5—C6—C11	−62.7 (4)	O2—C19—N1—C5	−8.3 (5)
C11—C6—C7—C8	0.9 (5)	C20—C19—N1—C5	170.5 (3)
C5—C6—C7—C8	−175.3 (3)	C2—C1—N1—C19	113.0 (3)
C6—C7—C8—C9	−1.1 (6)	C12—C1—N1—C19	−117.8 (3)
C7—C8—C9—C10	0.4 (7)	C2—C1—N1—C5	−48.8 (4)
C8—C9—C10—C11	0.4 (6)	C12—C1—N1—C5	80.3 (3)
C7—C6—C11—C10	−0.1 (5)	C6—C5—N1—C19	71.5 (3)
C5—C6—C11—C10	176.1 (3)	C4—C5—N1—C19	−162.4 (3)
C9—C10—C11—C6	−0.5 (6)	C6—C5—N1—C1	−125.4 (3)
N1—C1—C12—C13	−138.6 (3)	C4—C5—N1—C1	0.7 (4)

### Hydrogen-bond geometry (Å, °)

**Table:**

*D*—H···*A*	*D*—H	H···*A*	*D*···*A*	*D*—H···*A*
C2—H2A···O1^i^	0.97	2.56	3.458 (6)	154
C13—H13···O1^i^	0.93	2.51	3.404 (5)	161

Symmetry codes: (i) −*x*, −*y*+1, −*z*.
